# CD14/TLR4 priming potentially recalibrates and exerts anti-tumor efficacy in tumor associated macrophages in a mouse model of pancreatic carcinoma

**DOI:** 10.1038/srep31490

**Published:** 2016-08-11

**Authors:** Hridayesh Prakash, Vinod Nadella, Sandhya Singh, Hubertus Schmitz-Winnenthal

**Affiliations:** 1 Translational Immunology Division, German Cancer Research Center (DKFZ), Heidelberg, Germany; 2 National Center of Tumor diseases (NCT), Heidelberg, Germany; 3 Translational medicine Laboratory, School of life sciences, University of Hyderabad, Hyderabad, India; 4 Department of Animal Biology, School of Life sciences, University of Hyderabad, 500046, India; 5 Department of Visceral Surgery, University Medical Center, Heidelberg, Germany.

## Abstract

Pancreatic cancer is the fourth major cause of cancer related deaths in the world and 5 year survival is below 5%. Among various tumor directed therapies, stimulation of Toll-like receptors (TLR) has shown promising effects in various tumor models. However, pancreatic cancer cells frequently express these receptors themselves and their stimulation (TLR 2 and/or 4 particularly) within tumor microenvironment is known to potentially enhance tumor cell proliferation and cancer progression. Consistent stimulation of tumor associated macrophages (TAMs), in particular with tumor derived TLR ligand within the tumor microenvironment promotes cancer related inflammation, which is sterile, non-immunogenic and carcinogenic in nature. In view of this, recalibrating of TAM has the potential to induce immunogenic inflammation. Consistent with this, we provide experimental evidence for the first time in this study that priming of TAMs with TLR4 ligend (LPS) alone or in combination with IFN-γ not only recalibrates pancreatic tumor cells induced M2 polarization, but also confers anti-tumor potential in TAMs. Most interestingly, reduced tumor growth in macrophage depleted animals suggests that macrophage directed approaches are important for the management of pancreatic tumors.

Pancreatic cancer is one of the most aggressive forms of gastrointestinal cancers accounting for more than 25% cancer related deaths worldwide. While several factors including genetic mutation, diet, smoking and diabetes contribute to disease progression^1^,^2^,^3^, chronic inflammation which is manifested by recruitment of macrophages in particular to the pancreas, thought to be one major etiological factor increasing the risk for developing pancreatic cancer^4^,^5^. Symbiotic association of pancreatic tumor/satellite cells with tumor infiltrating macrophages (TAMs) supports metastatic spread of tumor cells during the early stages of carcinogenesis^6^,^7^,^8^. It is now believed that such tumor microenvironment not only confers a poor prognosis but also interferes with various tumor directed therapies. TAMs maintain a tumor beneficial microenvironment by secreting various factors including VEGF, TGF-β which only induce tumor cell growth but are also vital for maintenance of tumor vasculature. Both accumulation and density of TAMs within the tumor microenvironment are correlated with poor prognosis^9^ in cancer patients.

Under the influence of various cytokines/growth factors secreted by either tumor cells or macrophages during the course of tumorigenesis, the infiltrating iNOs+ regulatory M1 macrophages get strongly polarized to Arginase-1 & CD206+ M2 macrophages, which are immunosuppressive and promote tumor growth^10^,^11^,^12^,^13^ in multiple ways. Several reports have shown the accumulation of CD163^high^ and CD206+ TAMs in human and murine model of pancreatic tumors respectively^14^,^15^ leading to therapeutic challenges. Based on this, switching the M2 phenotype of TAMs to M1 regulatory macrophages^16^,^17^ has the potential to afford anti-tumor immunity.

The potential role of Low dose gamma irradiation (LDR) which we have recently proposed as a potential strategy for recalibrating TAMs towards M1^18^,^19^ in both murine insulinoma as well as in advanced stage pancreatic ductal adenocarcinoma (PDAC) patients, failed to induce anti-tumor immunity through irradiated macrophages against exponentially growing exocrine PancO2 tumor cells. Interestingly, priming of tumor induced macrophages with TLR4 agonist (LPS) alone or in combination with IFN-γ not only altered M2 polarization but also induced a strong anti-tumor immune reaction. Furthermore, our animal experimental data revealed the importance of macrophages in tumirogenesis. Taken together, our study confirms the significance of macrophage directed tumor therapies against aggressive cancer of pancreas.

## Results

### LPS signaling prevent tumor driven M2 reprogramming in macrophages.

**Figure 1:**
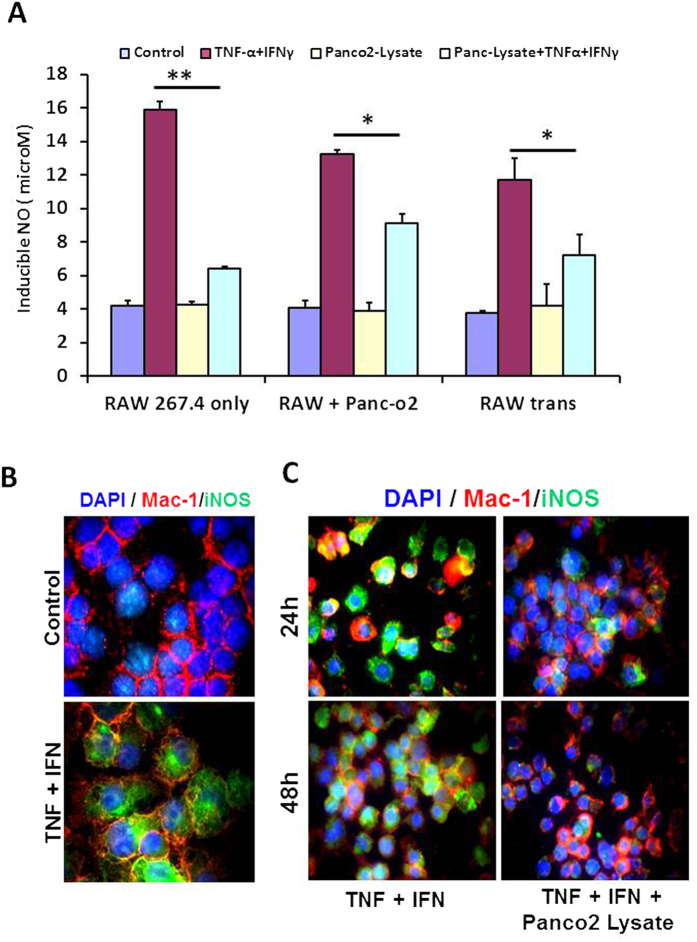
M1^dim^ polarization of M1^bright^ RAW macrophages by Panco2. (**A**) RAW264.7A murine MΦ were stimulated with Th1 cytokine with and without lysate prepared from PancO_2_ pancreatic tumor cell line (100 ng protein) both directly and indirectly and cultured for indicated time points. NO titer was quantified in the culture supernatants after 48 h by Griess reagent method. Shown here is the mean μM of NO ± SEM from three independent stimulation experiments. (*Indicate P < 0.05 and **P < 0.01). RAW macrophages were stimulated with TNF and IFN-γ in the absence (**B**) or presence (**C**) of Panco2 lysate, and the expression of iNOS proteins was visualized by immunofluorescence microscopy. Shown here are representative pictures obtained from two independent experiments.

**Figure 2:**
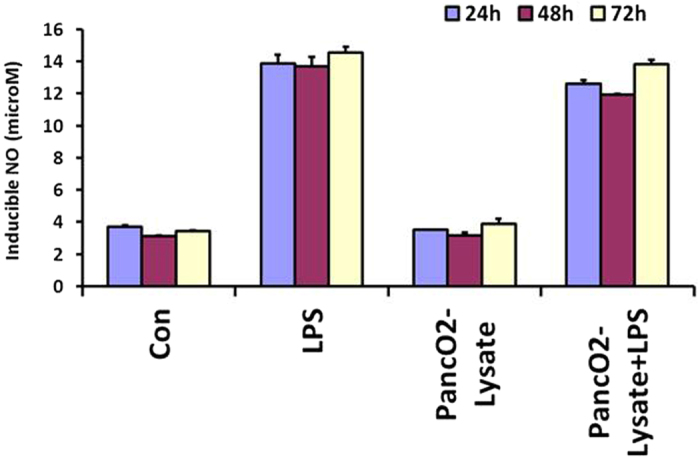
CD14/TLR4 stimulation mitigate tumor cell derived M1^dim^ programming in macrophages. RAW macrophages were cultured with and without PancO_2_ lysate in presence or absence of bacterial LPS and induced titer of NO was quantified in their culture supernatant at indicated time intervals. Shown here is the mean μM of NO ± SEM from three independent stimulation experiments.

**Figure 3:**
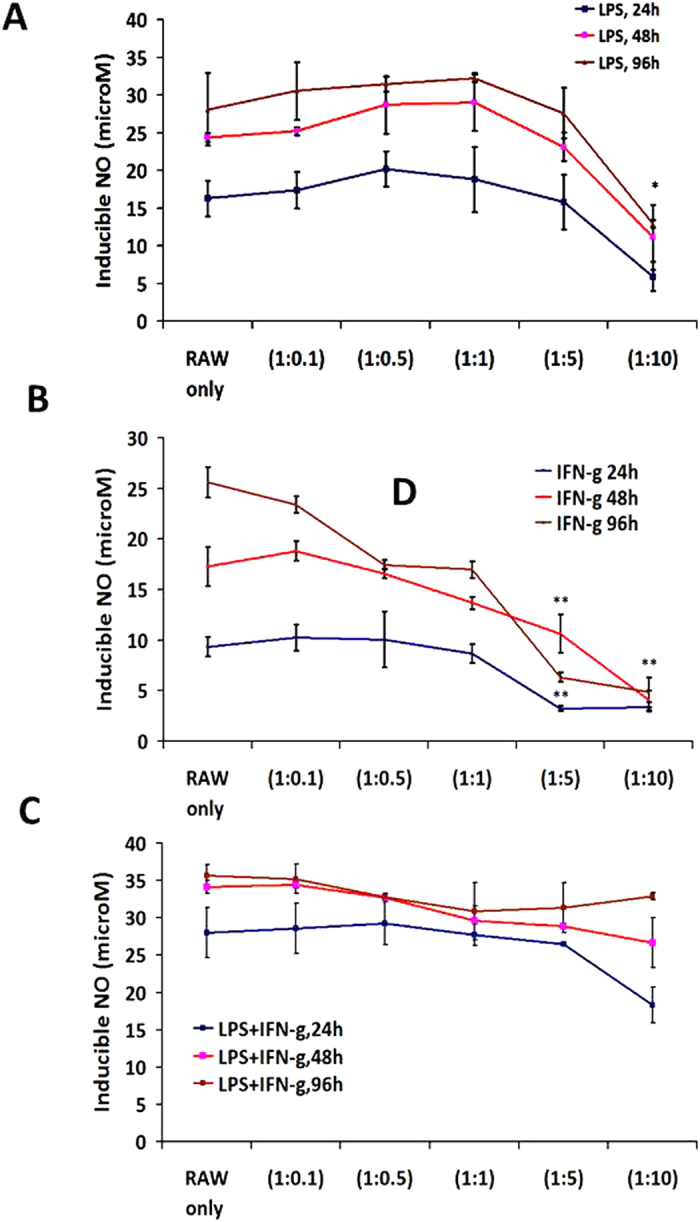
Co stimulation of RAW macrophages with CD14/TLR4 ligand (bacterial LPS) and IFN-γmitigate tumor cell derived M1^dim^ programming in iNOS+ and inflammatory macrophages. RAW macrophages and PancO_2_ co-cultures were stimulated with bacterial LPS (**A**) and IFN-γ (**B**) or together (**C**) and cultured for the indicated duration of time and their culture supernatant were quantified for NO titer. Shown here is the mean μM of NO ± SEM from five independent stimulation experiments. (*Indicate P < 0.05 and **P < 0.01).

**Figure 4:**
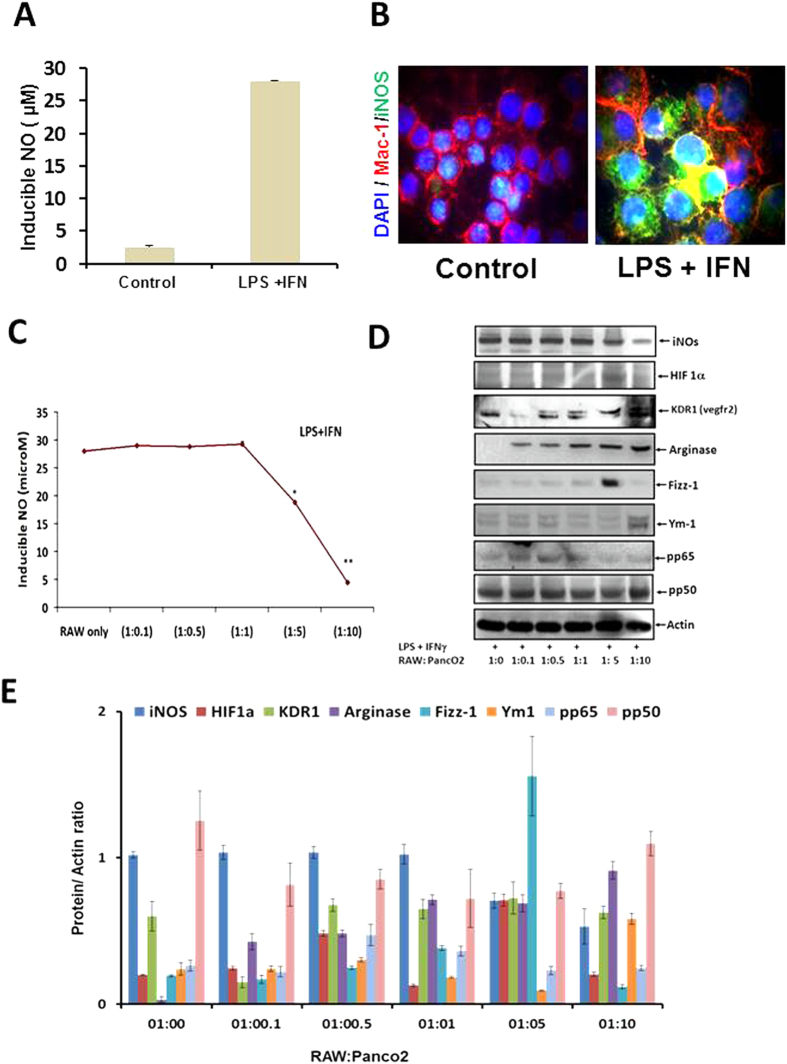
Transient stimulation of CD14/TLR4 and IFN signaling dose not mimic tumor cell derived M1^dim^ programming in M1 macrophages. RAW macrophages were stimulated with LPS/IFN and NO titer (**A**) and the expression of iNOS proteins was confirmed by immunofluorescence method (**B**) for showing M1 polarization of these macrophages against control. Shown here are the representative images of macrophages from several stimulations. (**C**) LPS + IFN stimulated RAW macrophages after culturing for 24 h were analyzed for the production of NO in their culture supernatant, washed and co-cultured further with varying amount of the tumor cells for another 72 h. Culture supernatants were collected and quantified for NO titer. Shown here is the mean μM of NO ± SEM from three independent stimulation experiments. (**D**) The co-cultures mentioned under **C** were lysed and analyzed for various M1 and M2 effector proteins by western blotting. Shown here is the representative blot from three independent experiments. (**E**) The western blots shown under (**D**) were quantified by densitometry. Shown here is the mean of protein/actin ratio ± SEM from three representative images with similar outcome. (*Indicate P < 0.05 and **P < 0.01).

Treatment of Th1 primed and iNOS+ RAW MΦ (Fig. 1A) with lysate prepared from well defined^20^ and exponentially growing (Suppl. Fig. 6) murine pancreatic ductal adenocarcinoma cell line (PancO2) significantly inhibited the generation of NO in their culture supernatants (Fig. 1A,B) and expression of iNOs proteins (Fig. 1C) in the Th1 primed macrophages demonstrating the M1^dim^ (NO^low^) programming of M1 effector macrophages by tumor lysate. These results for the first time demonstrate that tumor cells driven M2 programming is the characteristic of iNOS+ and NO producing M1 macrophages but not the resting and iNOS negative macrophages which neither produced nor changed their NO levels upon treatment with Panco2 lysate. This is quite interesting observation and suggest that M2 or M1^dim^ polarization is the characteristics of only activated and proinflammatory macrophages This is because of many intracellular signalling kinases like MAPK, STAT, and other critical intracellular proteins like Myd88, IRF, HIF1 and SHIP1, 2 proteins which work in concert only in activated macrophages and not in resting macrophages, contribute in NO production and in orchestration of Th1 (M1-) or Th2 (M2-) effector immune response. This motivated us to investigate whether interaction of MΦ with intact PancO_2_ cells will also have similar impact on MΦ retuning. To demonstrate this, TNF-α + IFN-γ stimulated RAW MΦ were co-cultured with PancO_2_ cells either directly or indirectly in trans-well chamber and NO levels in their culture supernatants were quantified. In line with above results, co-culture of macrophages (1:1 ratio) with PancO2 cells either directly or indirectly, consistently inhibited NO titre in cytokine differentiated iNOS+ MΦ (Fig. 1B) which produced copious amount of Th1 effector cytokines^18^ upon stimulation. However, the extent of inhibition was much lower by intact tumor cells in comparison to their lysate (Fig. 1A). Interestingly, PancO2 lysate could not inhibit LPS induced NO levels in these MΦ (Fig. 2) providing first evidence that TLR4 stimulation is decisive for the maintenance of M1 effector phenotype in Tumor induced MΦ (TIM). On this basis we argue whether such priming of TIM would confer similar retuning in MΦ when exposed to increasing tumor cell burden, as seen during tumor development. In order to demonstrate this, MΦ were co-cultured with increasing tumor cells (1:0.5–10) and quantified NO titers in these culture supernatants. Interestingly, co-culturing of un-stimulated and undifferentiated MΦ with increasing tumor cells also could not change the constitutive titer of NO (Suppl. Fig. 1) while inhibiting IFN-γ (Fig. 3B) or TNF-α+ IFN-γ (data not shown) induced NO level both in time and tumor cell number dependent manner. Surprisingly, neither LPS nor IFN stimulation on their own could prevent the drop in NO levels in the culture supernatants of these co-cultures up to 96 h post stimulation (Fig. 3A). However, treatment of these co-cultures with LPS and IFN-γ together not only enhanced NO titer significantly but also rescued NO levels even in highest R: P ratio group (Fig. 3C) up to 96 h, demonstrating the significance of LPS and IFN concerted signaling in M1 retuning of tumor induced M2 phenotype in macrophages. On these bases, we questioned whether priming of MΦ with LPS + IFN will sustain M1 effector phenotype upon their further challenge with tumor cells. To test this, RAW macrophages were primed with LPS + IFN-γ for 24 h to acquire M1 phenotype (Fig. 4A,B), washed and incubated with increasing number of PancO_2_ cells for another 72 h and NO titer was quantified in their culture supernatant. Contrary to our prediction, transient stimulation of RAW macrophages with LPS + IFN-γ could not mitigate PancO_2_ mediated loss of NO levels (Fig. 4C) in these co-cultures which correlated with a dose dependent decrease in iNOs expression, concurrent increase in expression of various M2 proteins (Fig. 4D,E), enhancement in of TGF-β and VEGF and its receptor KDR-1 (VEGFR2) levels (Suppl. Fig. 2A) and inhibition of M1 effector cytokines (Suppl. Fig. 2B,C) in their culture supernatant which altogether confirmed M2 polarization^10^,^11^,^21^,^22^ of tumor induced MΦ.

### TLR4 signaling and/or gamma irradiation both retune TAM

**Figure 5:**
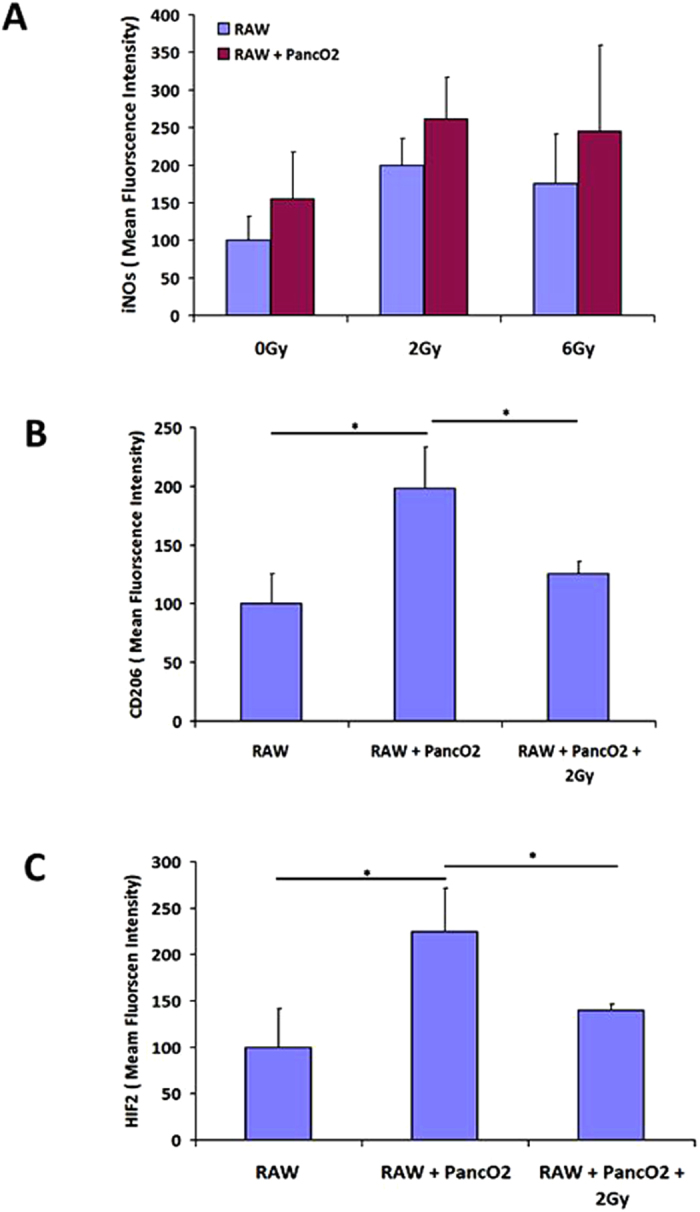
2 Gy gamma radiation down regulates M2 effector proteins in tumor fed macrophages. (**A**) RAW MΦ culture alone and their co-culture with Panc2 in (1:10 ratio) were irradiated with 2 Gy dose and analysed for the expression of iNOs (M1 effector protein) (**B**,**C**) and various M2 effector proteins after 48 h post irradiation. Shown here is the mean of MFI of the proteins ± SEM from five independent experiments. (*Indicate P < 0.05).

**Figure 6:**
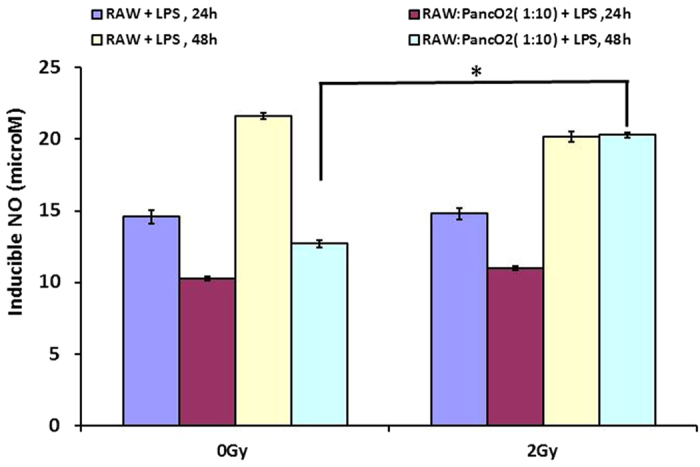
2 Gy and CD14 stimulation encounter M1^dim^ polarization of Tumor cells fed macrophages. The RAW-PancO_2_ co-cultures (1:10 ratio) were stimulated with LPS and cultured for indicated time intervals. Culture supernatants were analysed for NO titer. Data are represented as mean μM of NO ± SEM from three independent experiments. (*Indicate P < 0.05).

Among various neo-adjuvant tumor directed therapies, radiotherapy is a non-invasive strategy and used globally for inducing tumor immune rejection. Our recent study also demonstrated the influence of Gamma irradation on TLR4 induced M1 retuning of Tumor MΦ population^18^ from insulinoma bearing RipTag5 mice. We therefore analyzed whether Gamma irradiation and TLR4 co-stimulation would potentially retune Panco2 co-cultured RAW MΦ.To demonstrate this, RAW MΦ + PancO2 co-cultures were irradiated with 2 Gy dose of gamma irradiation and NO levels were quantified along with expression of intracellular M1/M2 proteins and various cytokines in their culture supernatants. Although, irradiation of RAW MΦ + PancO2 unstimulated co-cultures with 2 Gy enhanced expression of iNOs only marginally (Fig. 5A), it down regulated the expression of CD206 and HIF-2 significantly (Fig. 5B,C) over sham irradiated co-cultures. Time dependent increase in TNF-α titer, and rescue of IL-12p40 and p70 (Suppl. Fig. 3A,B), chemokines (Suppl. Fig. 3C) and decrease in Th2 effector cytokines (Suppl. Fig. 3D) in the irradiated co-cultures over sham suggested an overall depolarization of M2 MΦ. We next analyzed whether gamma irradiation of RAW MΦ + PancO2 co-culture would have synergistic impact on MΦ retuning, which was conferred by LPS. To this end, MΦ + PancO2 co-cultures were stimulated with LPS and irradiated with 2 Gy gamma radiation simultaneously and quantified NO titer. As expected, gamma irradiation of LPS stimulated MΦ + PancO2 co-cultures not only enhanced LPS induced NO levels synergistically but also rescued NO titers (Fig. 6). However, gamma irradiation could not modify NO titre in either IFN-γ alone or in LPS + IFN co-stimulated MΦ + PancO2 co-cultures over LPS only treated group, which could probably be due to TGF-β. iNOS proteins were barely enhanced in these co-cultures. Most interestingly, gamma irradiation of RAW MΦ + PancO2 stimulated co-cultures led to the reduction in the expression of CD206 (M2 differentiation marker) and HIF-2 proteins (Suppl. Fig. 4), drop in TGF-β and concurrent increase in IL-12 titer in LPS treated co-cultures (Suppl. Fig. 5) demonstrating the influence of gamma radiation and LPS driven M1, M2 reprogramming of tumor induced MΦ. Synergistic upregulation of CD14 receptors in co-cultures (Suppl. Fig. 4D) over RAW MΦ single cultures revealed the contribution of tumor cells in CD14 receptor expression and associated siganlling in driving macrophage polarization. This observation is in line with previous studies which demonstrated the role of tumor infiltration of CD14 receptor positive tumor as well as immune cells for the establishment of tumor supportive micro-environment which could be involved in M2 polarization of tumor infiltrated macrophages^23^,^24^,^25^,^26^ as well as in our experiments. These data for the first time demonstrate that TLR4 stimulation and/or gamma irradiation is paramount for retuning of^27^,^28^,^29^ tumor associated macrophages.

### TLR4 is critical for imparting anti-tumor potential in retuned TAM

Our recent findings have demonstrated the potential role of LDR in retuning TAM^18^,^19^ in neuroendocrine tumor of pancreas which failed to afford anti-tumor potential in irradiated macrophages against exponentially growing PancO2 tumor cells. On the basis of these observations that LDR in conjugation with LPS drive M1 re-programming of TAM, we strongly anticipated that TLR4/2 Gy driven retuning of TAM would also impart anti-tumor potential in these MΦ. In order to confirm this, survival of exponentially growing PancO_2_ tumor cells (Suppl. Fig. 6) was observed in presence of conditioned media (CM) from LPS or LPS + IFNγ stimulated MΦ. As expected, both of these condition media strongly inhibited Panco2 tumor cells growth in time dependent manner (Suppl. Fig. 7A–C). To further demonstrate the influence of gamma irradiation on anti-tumor potential of TLR4 and IFN-γ, RAW MΦ and Panco2 were co-cultured in trans well chambers. LPS+/− IFN-γ stimulated RAW cells with and without irradiation were maintained in the upper compartment and exponentially growing PancO_2_ tumor cells in the lower chamber to monitor their survival over the period of time. Interestingly and in contrast to our expectation, gamma irradiation could not improve LPS and/or IFNγ mediated tumor control (Suppl. Fig. 8) in tumor induced macrophages. Based on these observations, we were keen in translating our findings *in vivo*, and for that purpose PancO2 survival was analyzed under CM from thioglycolate elicited and MACS purified CD11b+ peritoneal MΦ from C57BL/6j mice. These cells were stimulated with Th1 effectors and irradiated. In line with earlier data from RAW macrophages, CM from LPS + IFN-γ treated CD11b+ primary macrophages also inhibited growth of PancO_2_ tumor cells strongly (Suppl. Fig. 9). Gamma irradiation of the primary macrophages once again and in line with RAW macrophages data could not improve LPS + IFN-γ mediated anti-tumor potential of CD11b+ macrophages. These observations indicated the radioresistant nature of PDAC tumor cells.

### Macrophages are important for the tumor development *in vivo*

**Figure 7:**
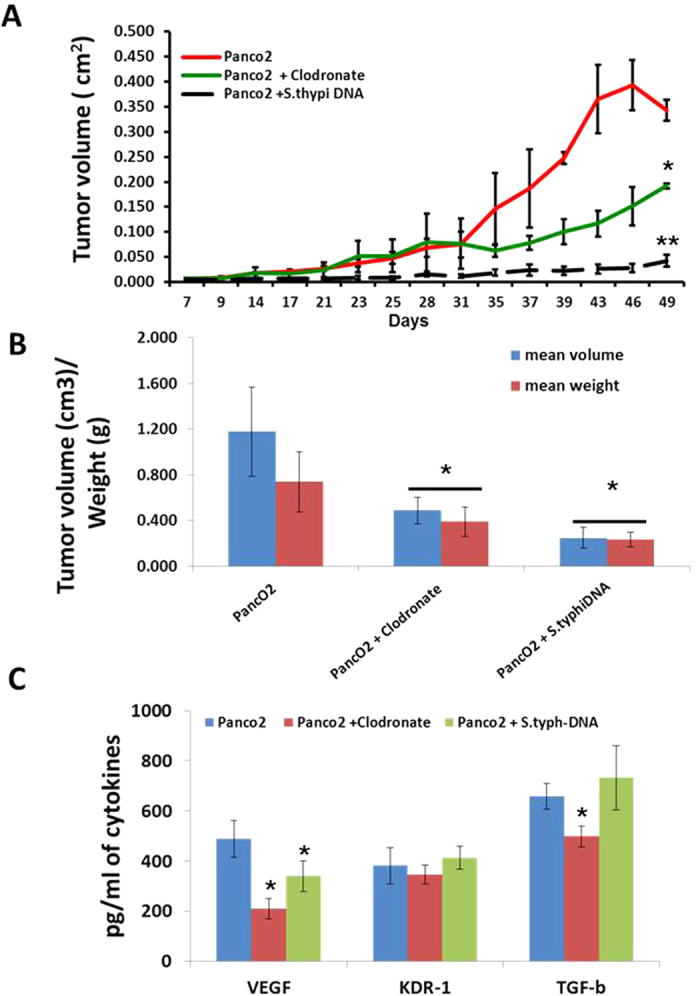
Macrophages are necessary for the tumor development. Macrophages were depleted in C57/BL6j mice by i.p. injection of clodronate liposomes. 1 × 10^4^ PancO_2_ tumor cells were implanted sub-cutaneously on both flanks of mice and tumor growth (**A**) was monitored over the period of 7 weeks. Shown here is the mean of tumor growth ± SEM from 10 mice. (**B**) Macroscopic and vascularized Panco2 tumors were excised at Day 49 and their physical parameters such as tumor volume and tumor weight were analyzed. (**C**) Intra-tumoral titer of VEGF and VEGFR2 and TGF-β were also quantified. Data are represented as mean of pg/ml of proteins ± SEM from 8 mice from PancO_2_ tumor alone and 4 mice each from clodronate and mice received empty plasmid from S. typhi DNA by oral route. (*Indicate P < 0.05 and **P < 0.01).

Accumulation of MΦ in the majority of tumors is correlated with poor prognosis and offers major hindrance in tumor directed therapies. Therefore, either their M1 retuning or depletion seems to entail therapeutic interventions in treating cancer patients. On these bases, we argue that depletion of peripheral MΦ population may bear impact on tumor growth. To test this hypothesis, peripheral macrophages in C57/Bl6 mice were depleted by weekly application of clodronate loaded liposomes which we have used in our recent study for depleting CD11b+ peripheral macrophages effectively^18^,^19^ and implanted PancO_2_ tumor cells subcutaneously to analyze the tumor growth over a period of time. As expected, depletion of MΦ not only reduced subcutaneous growth of pancreatic carcinoma (Fig. 7A,B) but also reduced key M2 effector cytokines titre (Fig. 7C) in these tumors^30^ demonstrating that MΦ are essential for tumor establishment and there depletion may offer treatment advantage. To translate the TLR4 driven tumor killing *in vitro*, mice were treated with empty plasmid from attenuated strain of S. typhimurium as a biological source of TLR-4 ligand and for inducing M1 effector immune response in tumor bearing mice. In line with our *in vitro* data, mice treated with this vaccine reduced the growth of implanted tumors. This was also accompanied with drop in VEGF titre (Fig. 7C), demonstrating potential retuning of tumor microenvironment. Most interestingly, the tumor growth in S. typhi DNA treated mice remained much lower than in mice depleted with MΦ (Fig. 7) which could be due to accumulation of M1 macrophages in these mice. Taken together our data for the first time revealed the significance of MΦ phenotyping and their role in immunity against exocrine tumors of pancreas.

## Discussion

Treatment strategies for pancreatic carcinoma are still in initial stages of development and those all explored could not prolong the median survival rate beyond few days to few weeks in cancer patients. This may be partly due to desmoplastic reactions manifested in stromal reactions which inhibit immune mediation rejection of tumors. Either under the influence of tumor microenvironment or by continuous uptake of dead tumor cells as a result of various anti-cancer drugs during treatment, peripheral or local MΦ gets strongly polarized from M1 MΦ towards M2 phenotype^22^,^31^. Such polarization is decisive for tumor progression and has been reported to be one key reason for failure of tumor directed immunotherapies. Thus, functional recalibrating of M2 to M1 phenotype may well prove decisive for an effective immunotherapy. Among various tumor directed interventions, radiotherapy has the potential to induce immune mediated tumor rejection. For an effective immunotherapy, the MΦ should not only acquire M1 characteristics but should also be able to kill tumor cells. In past, various approaches such as use of TLR agonists like CpG, CD40L etc. have been employed for boosting anti-tumor immunity^32^,^33^,^34^. Yet the success rate remain disappointing which could be because of other cells presence^35^, along with angiogenesis and tumor growth. Endogenous danger associated molecular pattern (DAMP’s) like heparan sulphate, heat shock proteins, HMGB1, hyaluronic acid are released from dying tumor cells which serve as ligands for various TLR’s within tumor microenvironment. Binding of these DAMP’s to their respective TLR’s trigger inflammation, promote tumor growth and immunosuppression^36^. Although these TLR ligands have equal chance of interacting with both MΦ as well as tumor cells in the established tumor microenvironment, abundance of tumor cells TLR signaling mainly activate tumor cells. Use of MΦ specific TLR’s in the immunosuppressive tumor microenvironment is still being at an early stage, selective activation of MΦ TLR’s by PAMPs^37^ has thus generated a lot of interest with the goal to selectively target TAMs. To this end, employing LPS alone or in combination with IFN-γ as an adjuvant to activate TLR4 signaling in MΦ effectively stabilized NO response in tumor induced MΦ and further assisted TAMs in controlling tumor cell growth. Our results justified using LPS as TLR4 ligand in order to induce M1 phenotype. Opposed to TLR4, LPS binds to CD14 receptor on myeloid cells, which drive strong Th1/Th17 signalling in MΦ and circulating dendritic cells. Intestingly, this receptor is mainly present on innate immune cells where their activation leads to the activation of Mitogen-activated protein kinase (MAPK), and others involved in protective inflammation as seen in LPS primed MΦ. We have recently demonstrated the importance and synergestic role of LDR in tumor infiltration of CD14+ MΦ in irraidated insulinomas, LPS induced expression of iNOS and generation of NO in MΦ from late stage insulimoa bearing mice^18^. Therefore we feel that upregulation of CD14 and associated signalling componants like IRAK, MyD88 and their activation^38^,^39^ could have contributed in M1 recalibrating of tumor induced MΦ. CD14+ immune cells are well known producers of TNF-α and NO which are the charactersitcs of M1 macrophages. Therefore we strongly believe that accumulation of these cells could have accounted for reduced tumor growth in S.Tyhpi-DNA vaccine administered mice. Beside altering the phenotype, selective depletion of TAMs also represents an alternative for the management of tumors as seen in breast cancer patients. Reduced PancO2 tumor growth in clodronate treated mice demonstrated that depleting TAM^30^,^40^ as a potentially easier path towards successful therapeutic intervention and management of aggressive cancers of the pancreas. Taken together, these results demonstrate the significance of macrophage directed therapy in controlling pancreatic cancer.

## Material and Methods

### Ethical statement

All methods mentioned in this section were carried out in “accordance” with the approved guidelines and experiments protocols were approved by the institutional Biosafety committee of German Cancer Research Centre. Animal experiments were authorized by the local government authority, Karlsruhe, Germany.

### Antibodies and reagents

RPMI 1640, Lipopolysaccharide (LPS), Penicillin Streptomycin solution, NaNO2, were purchased from Sigma (Taufkirchen, Germany). The Luminex kit was purchased from Bio-Rad Laboratories GmbH. Postfach 450133, D-80901 München, Germany, Sulphanilamide and N-(naphthyl) ethylene-diaminedihydrochloride were purchased from E-Merck (Darmstadt, Germany). Recombinant mouse TNF and IFN cytokines, Rabbit polyclonal anti-iNOs and anti-arginase-1 and anti-CD14 antibodies were purchased from BD transductions (Heidelberg, Germany). Rabbit anti-mouse pp65 (RelA), pp50 (nf-kb) antibodies were purchased from Cell signaling. Anti Actin antibody was purchased from Sigma and VEGFR2 antibody was purchased from Novus Biological. Rabbit anti-Ym-1 antibody was purchased from Stem cell technology; Fizz-1 antibody was purchased from Ab-cam. Anti-HIF-1 and 2 antibodies were purchased from Novus Biological Actin antibody was purchased from Sigma GmBH. Goat anti-rabbit HRP and Mouse HRP conjugated secondary antibodies were purchased from Sigma and Santa Cruz respectively.

### Cell culture experiments

RAW264.7A standard murine macrophage line was procured from ATCC and^41^,^42^ mouse pancreatic ductal adenocarcinoma cell line PancO2 was kind gift from Prof. Märten NCT, Heidelberg. These cells were co-cultured both directly as well indirectly. For direct co-culture experiments, RAW MΦs were seeded one day before for the adherence. Next day, cells were washed once with serum free media and PancO_2_ cells were seeded over the monolayer of RAW macrophages and these cells were cultured for one more night for the adherence of the tumor cells over macrophages. These cells were then stimulated with different stimuli for different time intervals as mentioned in the figures. For indirect cell culture, cells were cultured using cell culture insert whereby RAW cells were cultured over the membrane and PancO_2_ cells were cultured on the bottom. The RAW macrophages in the upper chamber or RAW and PancO_2_ direct co-cultures were stimulated with various innate stimuli or irradiated by using Co60 irradiator (Siemens, München, Germany) Co60 irradiator producing high energetic and low LET radiation. For evaluating the activation potential of irradiation and/or various stimulations, nitric oxide production was quantified in their culture supernatant by standard Griess reagent method. Various cytokines were quantified by luminex bead array (Bio-Rad) method. The effect of such activation of macrophages on PancO_2_ growth was analyzed by MTT dye method.

### Immunofluorescence staining

The cells were fixed with 4% PFA for 15 min and permeabilized by PBS-TritonX-100 (0.1%) for 5 min and blocked with TBS + 1% BSA + 1% sera from species of the respective secondary antibodies. After 2 washes with PBS, cells were incubated with primary antibodies overnight at 4 °C followed by incubation of cells with respective secondary antibodies (Alexa Fluor-488 for green and -569 for red color) for 1 h. Nuclei were stained with DAPI for 5 minutes. Stained cells were washed, mounted and analyzed with a fluorescent microscope (Axivort, Carl Zeiss) under 63× magnifications.

### Western analysis

The cells were lysed in RIPA buffer (50 mM Tris-HCl, pH 7.4, 150 mMNaCl, 2 mM EDTA, 1% Nonidet P-40, and protease inhibitor mixture) and sonicated. The lysate was centrifuged at 14,000 rpm for 20 min at 4 °C to separate the particulate fraction. Protein concentration was determined by BCA kit (Peirce). 20 μg protein per sample were separated on Nu-PAGE Bis-Tris Mini Gels system and blotted on PVDF membranes. Blots were blocked at room temperature for 30 minutes with 5% nonfat dry milk in TBS-T (20 mM Tris base, 137 mM NaCl, and 0.05% Tween 20, pH 7.5) and then incubated overnight at 4 °C with primary and subsequently with horseradish peroxidase-conjugated secondary antibodies. Blots were developed by ECL reagent (GE Healthcare). β-actin was used an internal loading control.

### Fluorimetric analysis

Various intracellular signaling proteins were measured by fluorimetric method. The RAW macrophages and PancO_2_ cells were co cultured as mentioned previously but in flat bottom black 96 well plates. These cells were irradiated and/stimulated and incubated for various time intervals up to 24 h. After incubation, the cell monolayers were washed once with PBS and fixed with 4% PFA for 10–15 minutes at RT and permeabilized with 0.1% Triton X for 5 minutes. The cell layers were then blocked in the PBS containing 1% BSA and 1% serum from the host of secondary antibodies. Next the cell layers were incubated with primary antibody at 4 °C overnight in dark. Next day, the cells were washed twice with 1X PBS. The cells were then incubated with Alexa Fluor conjugated secondary antibodies (In-vitrogen) for 1 h at RT in dark. The cell layers were washed again twice with PBS. The plates were read at 488 nm for Alexa Fluor -488 labeled antibodies or at 594 nm for Alexa-Fluor 594 labeled antibodies respectively with a reference wavelength of 630 using a 96 well fluorimeter (TECAN infinite 200 PRO systems). Each well was read at multiple fields for the uniformity and the mean fluorescence intensities of stained cells from each well were calculated.

### Subcutaneous tumor experimentation

8–10 weeks old C57/Bl6j mice were used for this study. In order to deplete the macrophages, these mice were injected clodronate liposomes intraperitoneally 2–3 which we recently reported^18^,^19^ as one of effective strategy of depleting macrophages in mice. These mice were injected 1.0 × 10^5^ PancO_2_ cells in 100 μL sterile NaCl, 0.9% (m/v), intracutaneously in both left and right flank of mice and tumor growth was monitored for the indicated time interval. For delivering the TLR ligands in these mice, these mice were treated orally with empty plasmid from attenuated strain of S. typhimurium as biological source of delivering TLR-4 ligand. Physical examination of tumor size and volume was done at regular intervals.

### Statistical analysis

Data sets were calculated and analyzed by Student’s t test. *P < 95% and **P < 99% confidence interval were considered significant.

## Additional Information

**How to cite this article**: Prakash, H. *et al*. CD14/TLR4 priming potentially recalibrates and exerts anti-tumor efficacy in tumor associated macrophages in a mouse model of pancreatic carcinoma. *Sci. Rep.* **6**, 31490; doi: 10.1038/srep31490 (2016).

## Supplementary Material

Supplementary Information

## References

[b1] Cappellani A. . Diet and pancreatic cancer: many questions with few certainties. Eur. Rev. Med. Pharmacol. Sci. 16, 192–206 (2012). 22428470

[b2] Evans J. P. . Familial pancreatic adenocarcinoma: association with diabetes and early molecular diagnosis. J. Med. Genet. 32, 330–335 (1995). 7616537 10.1136/jmg.32.5.330 PMC1050425

[b3] Li D. . DNA repair gene polymorphisms and risk of pancreatic cancer. Clin. Cancer Res. 15, 740–746 (2009). 19147782 10.1158/1078-0432.CCR-08-1607 PMC2629144

[b4] Teich N. & Mossner J. Hereditary chronic pancreatitis. Best. Pract. Res. Clin. Gastroenterol. 22, 115–130 (2008). 18206817 10.1016/j.bpg.2007.10.019

[b5] Jura N., Archer H. & Bar-Sagi D. Chronic pancreatitis, pancreatic adenocarcinoma and the black box in-between. Cell Res. 15, 72–77 (2005). 15686632 10.1038/sj.cr.7290269

[b6] Menen R. S. . Tumor-educated Macrophages Promote Tumor Growth and Peritoneal Metastasis in an Orthotopic Nude Mouse Model of Human Pancreatic Cancer. In Vivo 26, 565–569 (2012). 22773569

[b7] Esposito I. . Inflammatory cells contribute to the generation of an angiogenic phenotype in pancreatic ductal adenocarcinoma. J. Clin. Pathol. 57, 630–636 (2004). 15166270 10.1136/jcp.2003.014498 PMC1770337

[b8] Gardian K., Janczewska S., Olszewski W. L. & Durlik M. Analysis of Pancreatic Cancer Microenvironment: Role of Macrophage Infiltrates and Growth Factors Expression. J. Cancer 3, 285–291 (2012). 22773932 10.7150/jca.4537 PMC3390598

[b9] Siveen K. S. & Kuttan G. Role of macrophages in tumour progression. Immunol. Lett. 123, 97–102 (2009). 19428556 10.1016/j.imlet.2009.02.011

[b10] Allavena P., Sica A., Solinas G., Porta C. & Mantovani A. The inflammatory micro-environment in tumor progression: the role of tumor-associated macrophages. Crit Rev. Oncol. Hematol. 66, 1–9 (2008). 17913510 10.1016/j.critrevonc.2007.07.004

[b11] Sica A., Allavena P. & Mantovani A. Cancer related inflammation: the macrophage connection. Cancer Lett. 267, 204–215 (2008). 18448242 10.1016/j.canlet.2008.03.028

[b12] Solinas G. . Tumor-conditioned macrophages secrete migration-stimulating factor: a new marker for M2-polarization, influencing tumor cell motility. J. Immunol. 185, 642–652 (2010). 20530259 10.4049/jimmunol.1000413

[b13] Pollard J. W. Tumour-educated macrophages promote tumour progression and metastasis. Nat. Rev. Cancer 4, 71–78 (2004). 10.1038/nrc1256 14708027

[b14] Kurahara H. . Significance of M2-polarized tumor-associated macrophage in pancreatic cancer. J. Surg. Res. 167, e211–e219 (2011). 10.1016/j.jss.2009.05.026 19765725

[b15] Kurahara H. . M2-Polarized Tumor-Associated Macrophage Infiltration of Regional Lymph Nodes Is Associated With Nodal Lymphangiogenesis and Occult Nodal Involvement in pN0 Pancreatic Cancer. Pancreas (2012). 10.1097/MPA.0b013e318254f2d1 22699204

[b16] Ma J. . The M1 form of tumor-associated macrophages in non-small cell lung cancer is positively associated with survival time. BMC. Cancer 10, 112 (2010). 10.1186/1471-2407-10-112 PMC2851690 20338029

[b17] Ohri C. M., Shikotra A., Green R. H., Waller D. A. & Bradding P. Macrophages within NSCLC tumour islets are predominantly of a cytotoxic M1 phenotype associated with extended survival. Eur. Respir. J. 33, 118–126 (2009). 10.1183/09031936.00065708 19118225

[b18] Prakash H. . Low doses of gamma irradiation (LDR) potentially modifies immunosuppressive tumor microenvironment by retuning tumor-associated macrophages (TAM): lesson from insulinoma. Carcinogenesis (2016). 10.1093/carcin/bgw007 26785731

[b19] Klug F. . Low-Dose Irradiation Programs Macrophage Differentiation to an iNOS(+)/M1 Phenotype that Orchestrates Effective T Cell Immunotherapy. Cancer Cell 24, 589–602 (2013). 24209604 10.1016/j.ccr.2013.09.014

[b20] Corbett T. H. . Induction and chemotherapeutic response of two transplantable ductal adenocarcinomas of the pancreas in C57BL/6 mice. Cancer Res. 44, 717–726 (1984). 6692374

[b21] Ye S. . The E3 ubiquitin ligase Nrdp1 promotes M2 macrophage polarization by ubiquitinating and activating transcription factor C/EBPbeta. J. Biol. Chem. (2012). 10.1074/jbc.M112.383265 PMC3411012 22707723

[b22] Benoit M., Desnues B. & Mege J. L. Macrophage polarization in bacterial infections. J. Immunol. 181, 3733–3739 (2008). 18768823 10.4049/jimmunol.181.6.3733

[b23] Cheah M. T. . CD14-expressing cancer cells establish the inflammatory and proliferative tumor microenvironment in bladder cancer. Proc. Natl. Acad. Sci. USA 112, 4725–4730 (2015). 25825750 10.1073/pnas.1424795112 PMC4403197

[b24] Maeda Y. . Myeloid Differentiation Factor 88 Signaling in Bone Marrow-Derived Cells Promotes Gastric Tumorigenesis by Generation of Inflammatory Microenvironment. Cancer Prev. Res. (Phila) 9, 253–263 (2016). 10.1158/1940-6207.CAPR-15-0315 26888865

[b25] Schwacha M. G., Chaudry I. H. & Alexander M. Regulation of macrophage IL-10 production postinjury via beta2 integrin signaling and the P38 MAP kinase pathway. Shock 20, 529–535 (2003). 10.1097/01.shk.0000095059.62263.56 14625477

[b26] Brophy V. H. & Sibley C. H. Expression of CD14 corrects the slow response to lipopolysaccharide in the 1B8 mutant of the B cell lymphoma 70Z/3. Immunogenetics 47, 196–205 (1998). 9435337 10.1007/s002510050348

[b27] Meng J., Gong M., Bjorkbacka H. & Golenbock D. T. Genome-wide expression profiling and mutagenesis studies reveal that lipopolysaccharide responsiveness appears to be absolutely dependent on TLR4 and MD-2 expression and is dependent upon intermolecular ionic interactions. J. Immunol. 187, 3683–3693 (2011). 10.4049/jimmunol.1101397 PMC3178671 21865549

[b28] Moreno C. . Lipopolysaccharide needs soluble CD14 to interact with TLR4 in human monocytes depleted of membrane CD14. Microbes. Infect. 6, 990–995 (2004). 10.1016/j.micinf.2004.05.010 15345230

[b29] Lien E. . Toll-like receptor 4 imparts ligand-specific recognition of bacterial lipopolysaccharide. J. Clin. Invest 105, 497–504 (2000). 10683379 10.1172/JCI8541 PMC289161

[b30] Gazzaniga S. . Targeting tumor-associated macrophages and inhibition of MCP-1 reduce angiogenesis and tumor growth in a human melanoma xenograft. J. Invest Dermatol. 127, 2031–2041 (2007). 10.1038/sj.jid.5700827 17460736

[b31] Matuschek A. . Analysis of parathyroid graft rejection suggests alloantigen-specific production of nitric oxide by iNOS-positive intragraft macrophages. Transpl. Immunol. 21, 183–191 (2009). 19409993 10.1016/j.trim.2009.04.004

[b32] Lapteva N. . Enhanced activation of human dendritic cells by inducible CD40 and Toll-like receptor-4 ligation. Cancer Res. 67, 10528–10537 (2007). 10.1158/0008-5472.CAN-07-0833 17974997

[b33] Nierkens S. . Immune adjuvant efficacy of CpG oligonucleotide in cancer treatment is founded specifically upon TLR9 function in plasmacytoid dendritic cells. Cancer Res. 71, 6428–6437 (2011). 21788345 10.1158/0008-5472.CAN-11-2154 PMC3653311

[b34] Kim B. S. . Inflammatory mediators are inhibited by a taurine metabolite in CpG oligodeoxynucleotide and IFN-r activated macrophage cell line. J. Drugs Dermatol. 12, 551–557 (2013). 23652950

[b35] Basith S., Manavalan B., Yoo T. H., Kim S. G. & Choi S. Roles of toll-like receptors in cancer: a double-edged sword for defense and offense. Arch. Pharm. Res. 35, 1297–1316 (2012). 22941474 10.1007/s12272-012-0802-7

[b36] Sato Y., Goto Y., Narita N. & Hoon D. S. Cancer Cells Expressing Toll-like Receptors and the Tumor Microenvironment. Cancer Microenviron. 2 Suppl. 1, 205–214 (2009). 10.1007/s12307-009-0022-y PMC2756339 19685283

[b37] Tsan M. F. Toll-like receptors, inflammation and cancer. Semin. Cancer Biol. 16, 32–37 (2006). 10.1016/j.semcancer.2005.07.004 16153858

[b38] Islam M. A. . Alveolar macrophage phagocytic activity is enhanced with LPS priming, and combined stimulation of LPS and lipoteichoic acid synergistically induce pro-inflammatory cytokines in pigs. Innate. Immun. 19, 631–643 (2013). 10.1177/1753425913477166 23608822

[b39] Tachado S. D. . MyD88-dependent TLR4 signaling is selectively impaired in alveolar macrophages from asymptomatic HIV+ persons. Blood 115, 3606–3615 (2010). 10.1182/blood-2009-10-250787 PMC2867269 20197549

[b40] Zeisberger S. M. . Clodronate-liposome-mediated depletion of tumour-associated macrophages: a new and highly effective antiangiogenic therapy approach. Br. J. Cancer 95, 272–281 (2006). 16832418 10.1038/sj.bjc.6603240 PMC2360657

[b41] Shevchenko I. . Low-dose gemcitabine depletes regulatory T cells and improves survival in the orthotopic Panc02 model of pancreatic cancer. Int. J. Cancer 133, 98–107 (2013). 10.1002/ijc.27990 23233419

[b42] Guleng B. . Blockade of the stromal cell-derived factor-1/CXCR4 axis attenuates *in vivo* tumor growth by inhibiting angiogenesis in a vascular endothelial growth factor-independent manner. 65, 5864–5871 (2005). 10.1158/0008-5472.CAN-04-3833 15994964

