# Supplementary material for: CD14/TLR4 priming potentially recalibrates and exerts anti-tumor efficacy in tumor associated macrophages in a mouse model of pancreatic carcinoma
Source: Sci Rep. 2016 Aug 11;6:31490. doi: 10.1038/srep31490 (PMC4980608; doi:10.1038/srep31490)

**Supportive Information:**

**CD14/ TLR4 priming potentially recalibrates and exerts anti-tumor efficacy in tumor associated macrophages in a mouse model of pancreatic carcinoma.**

Hridayesh Prakash, Vinod Nadella, Sandhya Singh and Hubertus Schmitz-Winnenthal

**Legend to supplementary figures:**

**Suppl Figure1- Panco2 tumor cells does not modify NO in resting Macrophages**

RAW macrophages were co cultured with increasing number of PancO_2_ and their supernatant were collected to measure NO by Griess reagent method. Shown here is the mean µM of NO ± SEM from five independent stimulation experiments.

**Suppl Figure 2- Pancreatic carcinoma cells induces angiogenic response in the inflammatory macrophages**

The supernatant from RAW macrophages and PancO_2_ co-culture as described under **Fig. 2E** were collected. These were analyzed for Key M2 effector cytokines **(A),** anti-tumor cytokines **(B-D)** and several interleukins **(E, F)** after 24h post culture. Shown here is the pg of proteins ± SEM from 3 independent analyses. (* indicate P < 0.05 and ** P < 0.01)

**Suppl Figure 3-** **2Gy dose enhances the secretion of TNF and inhibit IL-6 in M2 rich co-culture of RAW and PancO2**

RAW- PancO_2_ co-cultures were irradiated with 2Gy and the titer of various cytokines were quantified at 24h **(A)** and 48h **(B)** post irradiation in their culture supernatant by Luminex method as described in the text. **C.** Various chemokines were also quantified in above culture supernatant at indicated time intervals **D**. The same RAW-PancO2 co-culture supernatant was also analyzed for the various Interleukins by Luminex method at indicated time interval. Data are represented as mean of pg of cytokines/ interleukins ± SEM from 2 independent experiments. (* indicate P < 0.05)

**Suppl Figure 4-** **Synergetic stimulation ofCD14/TLR4 and IFN signaling mitigate tumor cell derived M1^dim^ programming in M1 macrophages**

RAW macrophages and PancO_2_ co cultures (1:10 ratio) were stimulated with IFN-γ **(A)** with and without bacterial LPS **(B)** and cultured for the indicated duration of time and their culture supernatant NO titer was quantified at indicated time intervals. Shown here is the mean µM of NO ± SEM from five independent stimulation experiments. (* indicate P < 0.05 )

**Suppl Figure 5-** **2Gy and CD14/IFN stimulation modify M2 re-programming in tumor cell fed macrophages**

Culture supernatant of co-cultures were collected and analyzed for the secretion of Th1 and Th2 effector cytokines by luminex assay. Data are represented as mean of pg of cytokines/ interleukins ± SEM from 2 independent experiments. (* indicate P < 0.05 and ** P < 0.01)

**Suppl. Figure 6. PancO2 cell grown aggressively**

Log culture of Panco2 cell were seeded in 96 well plate at 1x 10^4^ cell per well and their growth was analyzed by MTT based method. Shown here is the ( % growth data) obtained from 5 independent experiments.

**Suppl Figure 7. CD14 and IFN stimulation render macrophages tumoricidal**

RAW264.7A murine macrophage were co-cultured with Panco2 in 1:10 ratio in trans-well chamber where The macrophages were placed in the upper chamber and Panco2 were placed underneath and macrophages were stimulated with various innate stimuli and survival of PancO2 cell was monitored at indicated time intervals. Data are represented as mean ± SEM of the survival of PancO2 cells from 3 independent growth experiments. (* indicate P < 0.05 and ** P < 0.01)

**Suppl Figure 8- Stimulation of CD14 alone in tumor fed macrophages is sufficient to control tumor growth**

RAW single and RAW : PancO2 direct co-culture were irradiated with 2Gy dose and stimulated with LPS and IFN and their condition media were collected at 48h and PancO_2_ cells were grown in condition media and their survival was monitored for **24h(A), 48h (B)** and **72h (C).** Shown here is the mean ± SEM of the survival of PancO2 cells from 3 independent growth experiments. (* indicate P < 0.05 and ** P < 0.01)

**Suppl Figure 9-** **CD14 and IFN concerted signalling induces anti-tumor response in mouse primary macrophages**

CD11b+/Gr-1- primary macrophages were purified from C57/BL6j mice using MACS based method. These were irradiated and stimulated as indicated and their condition media were collected after 48h . 1x10^4^ (PancO_2_) cells were cultured in presence of the conditioned medium prepared form LPS+IFN or TNF+IFN stimulated the Panco2 tumor cell growth was monitored for 24h (A), 48h(B) and 96h (C). Shown here is the mean ± SEM of the survival of PancO2 cells from 3 independent growth experiments. (* indicate ** P < 0.01)


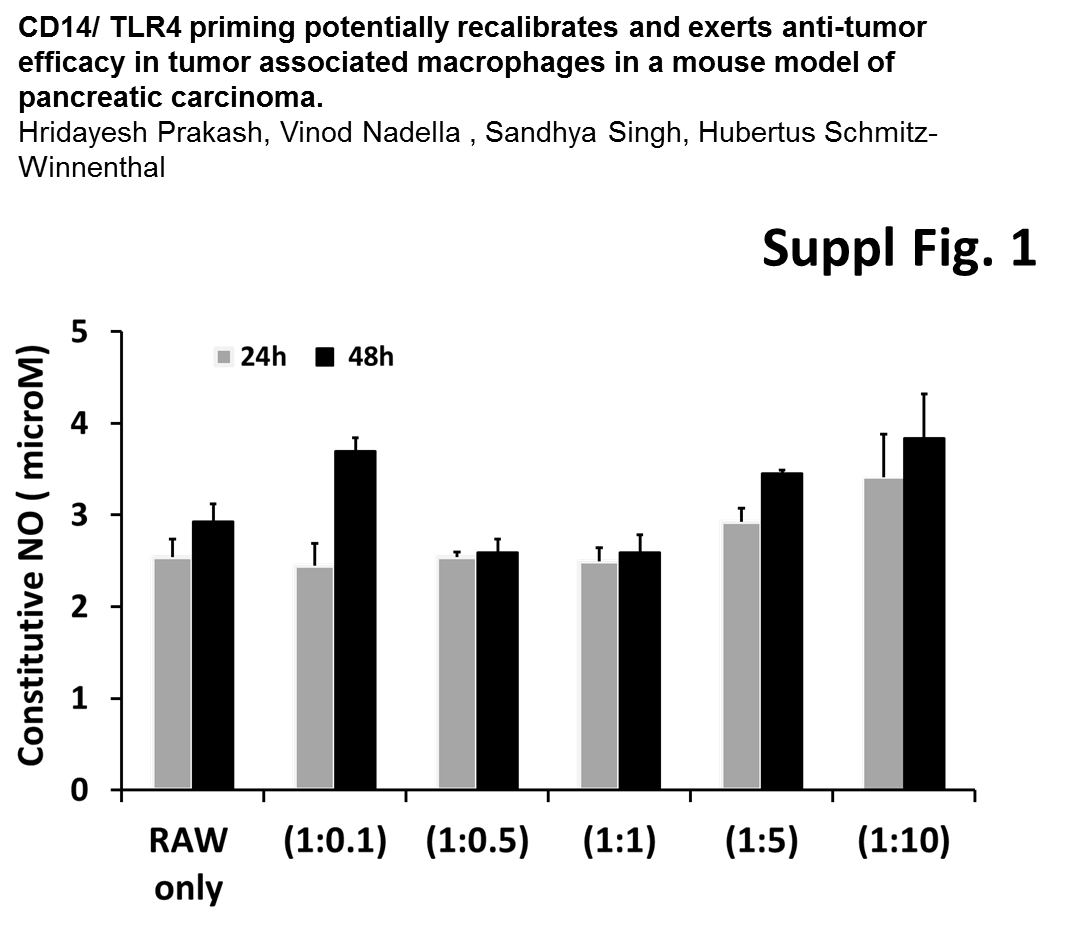


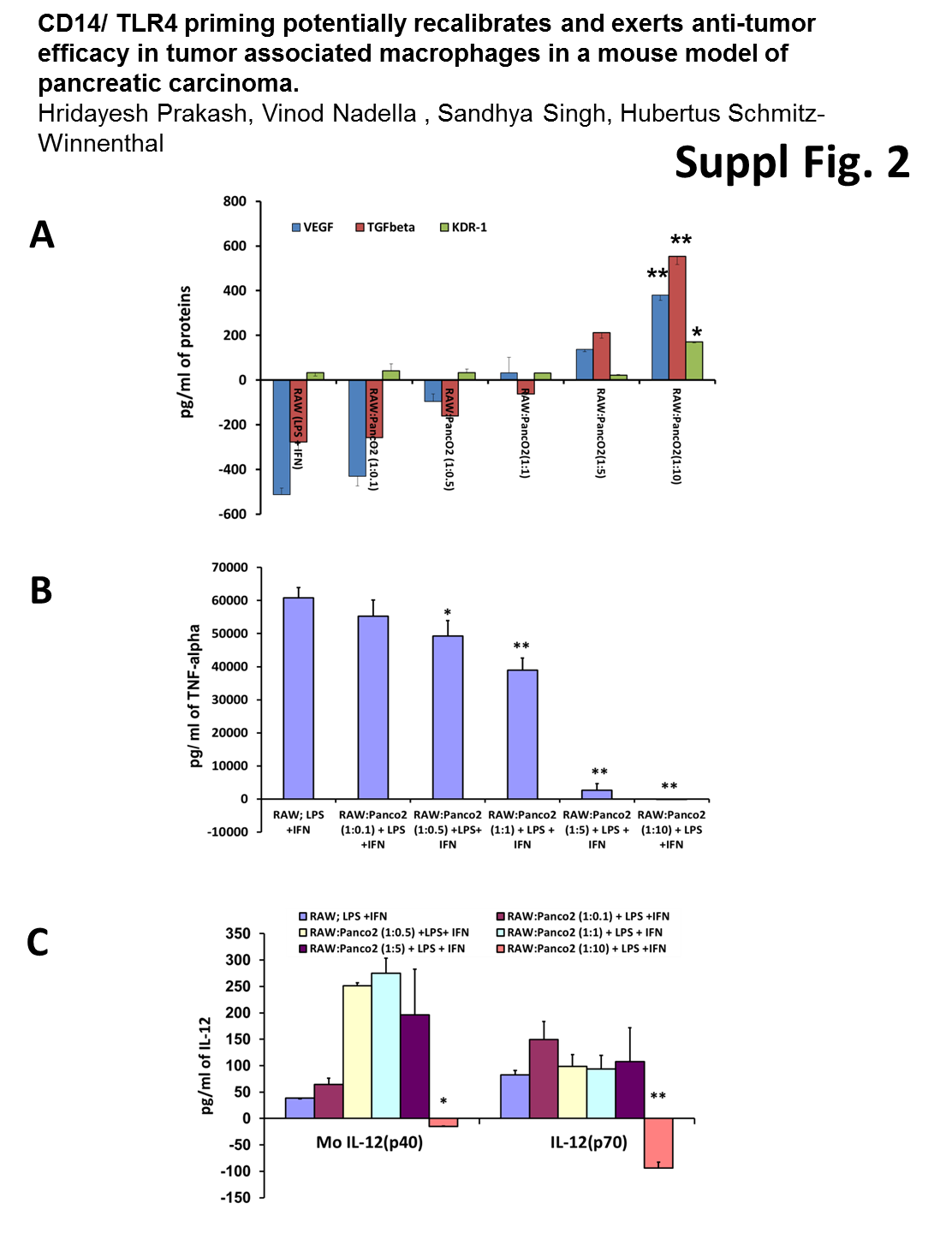


**B**

**C**

**D**

**A**


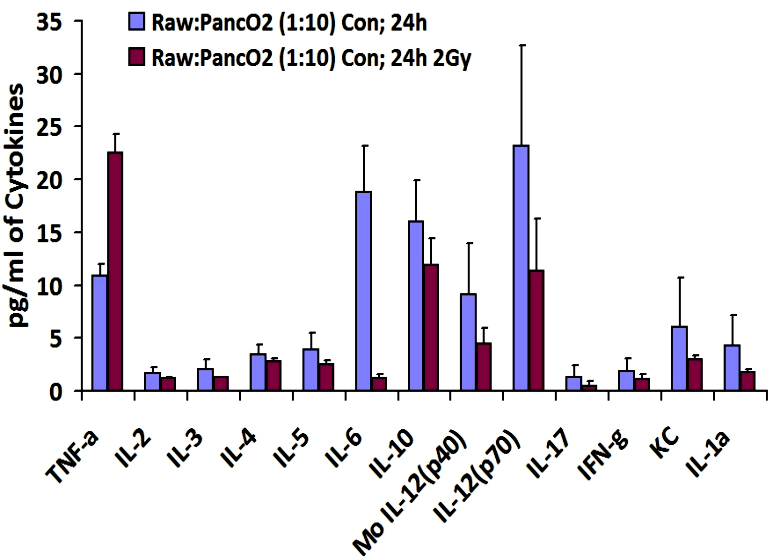

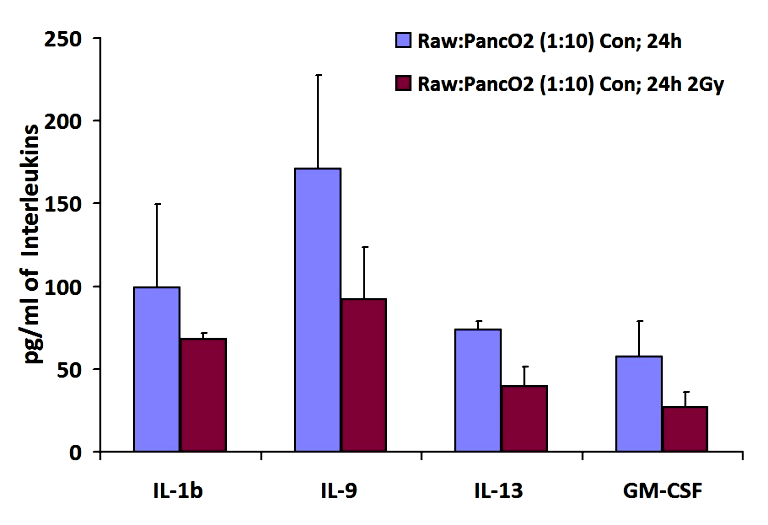

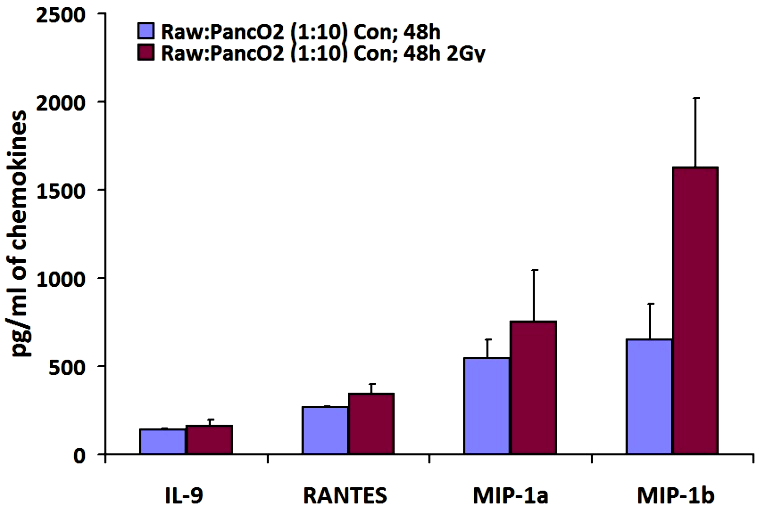


**Suppl Fig. 3**

*****

*****

**CD14/ TLR4 priming potentially recalibrates and exerts anti-tumor efficacy in tumor associated macrophages in a mouse model of pancreatic carcinoma.**

Hridayesh Prakash, Vinod Nadella , Sandhya Singh, Hubertus Schmitz-Winnenthal


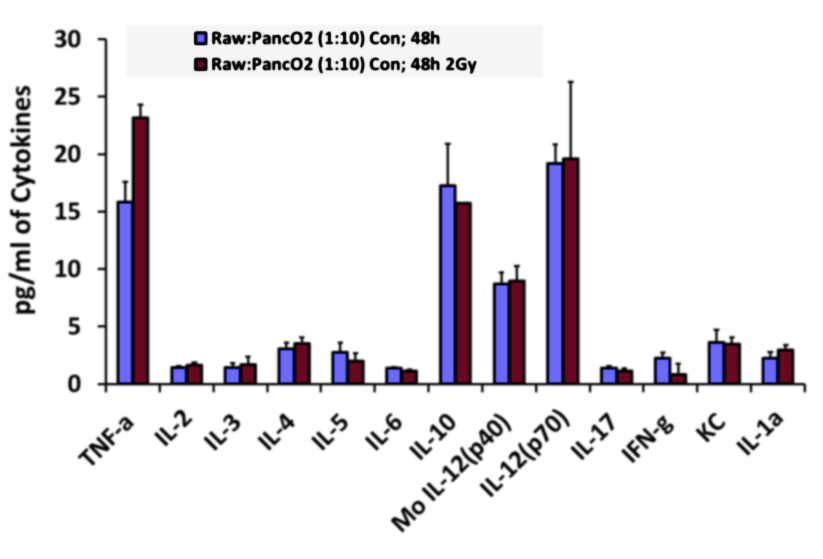


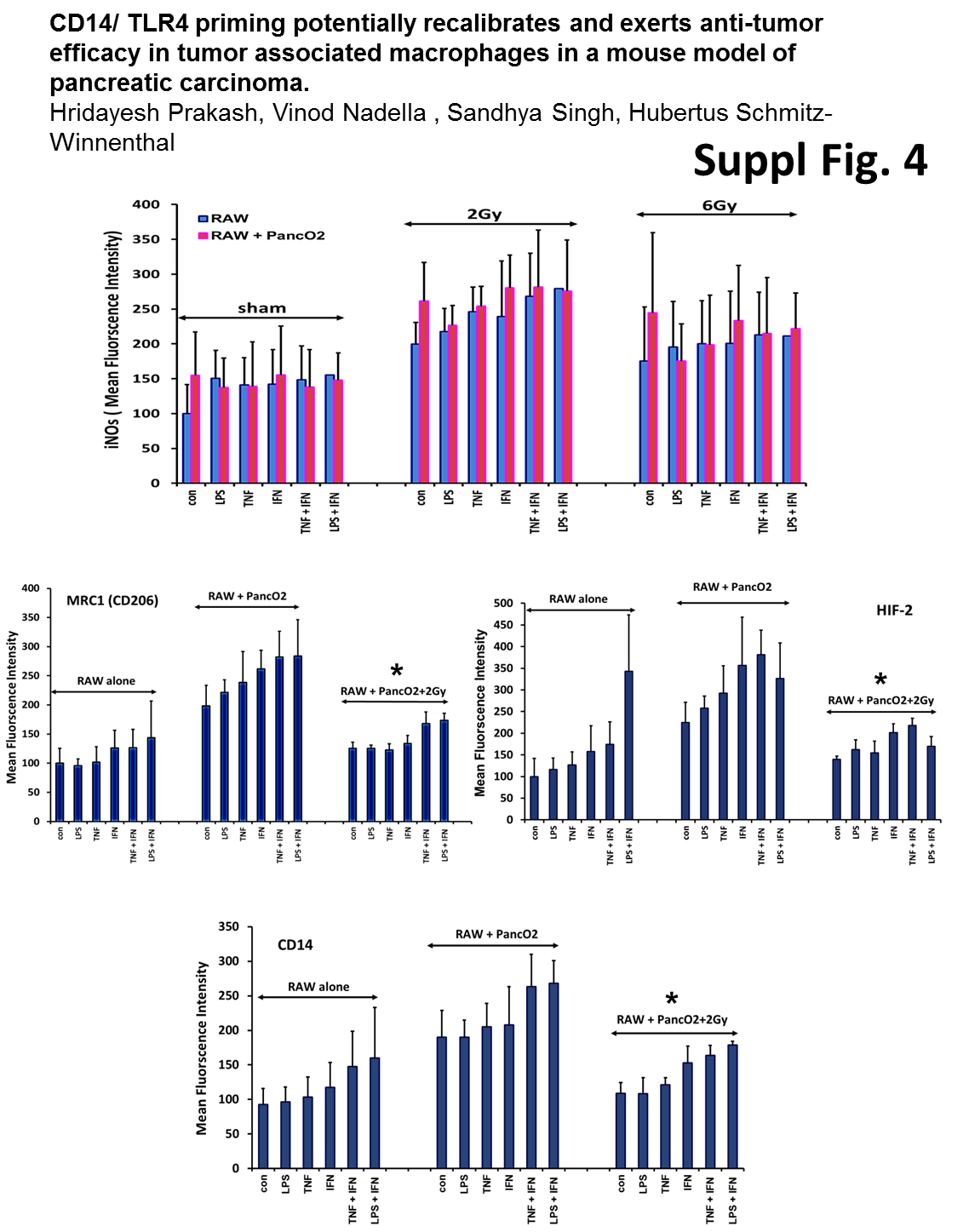


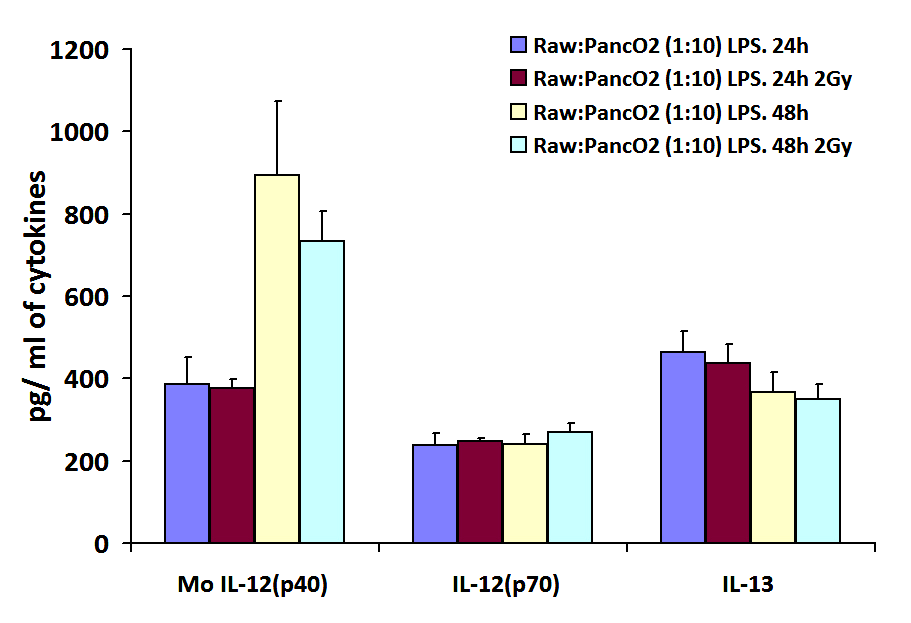

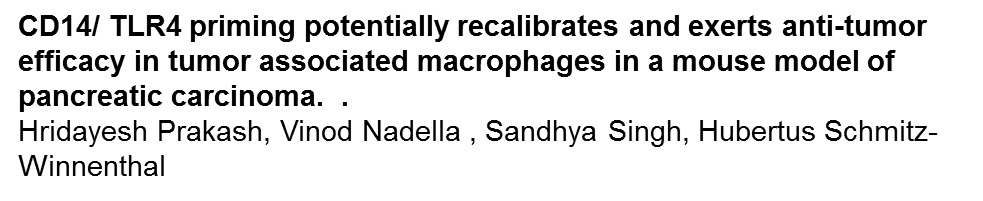

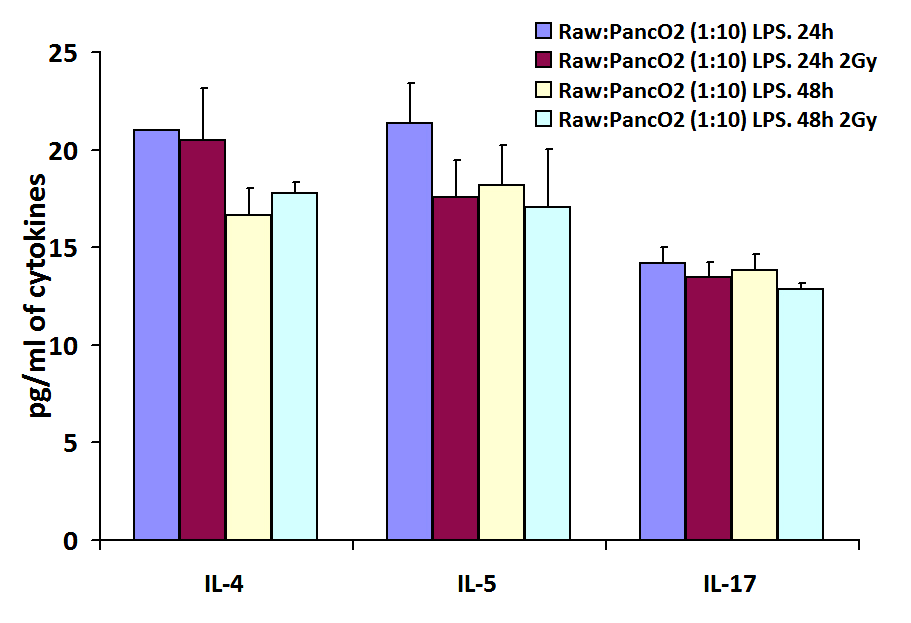

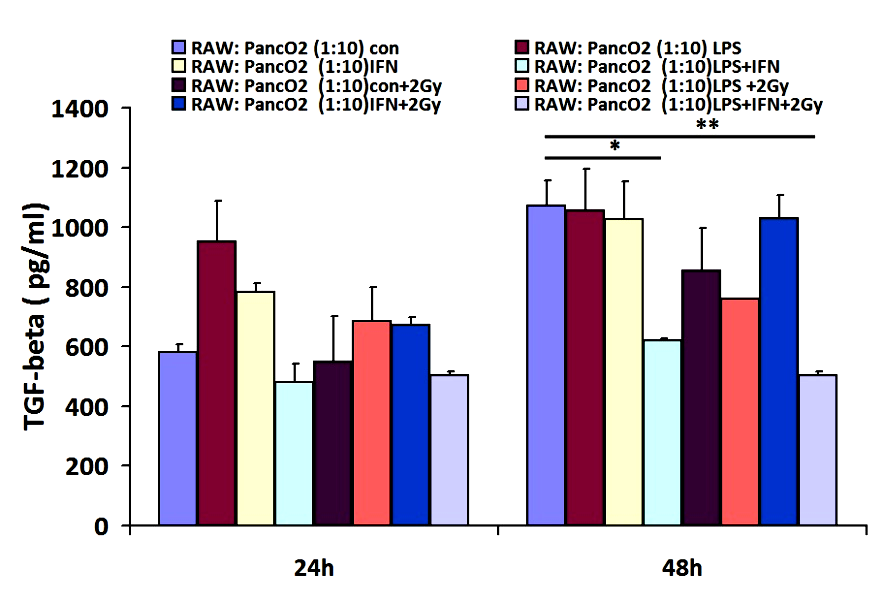


**B**

**C**

**A**

**Suppl Fig. 5**


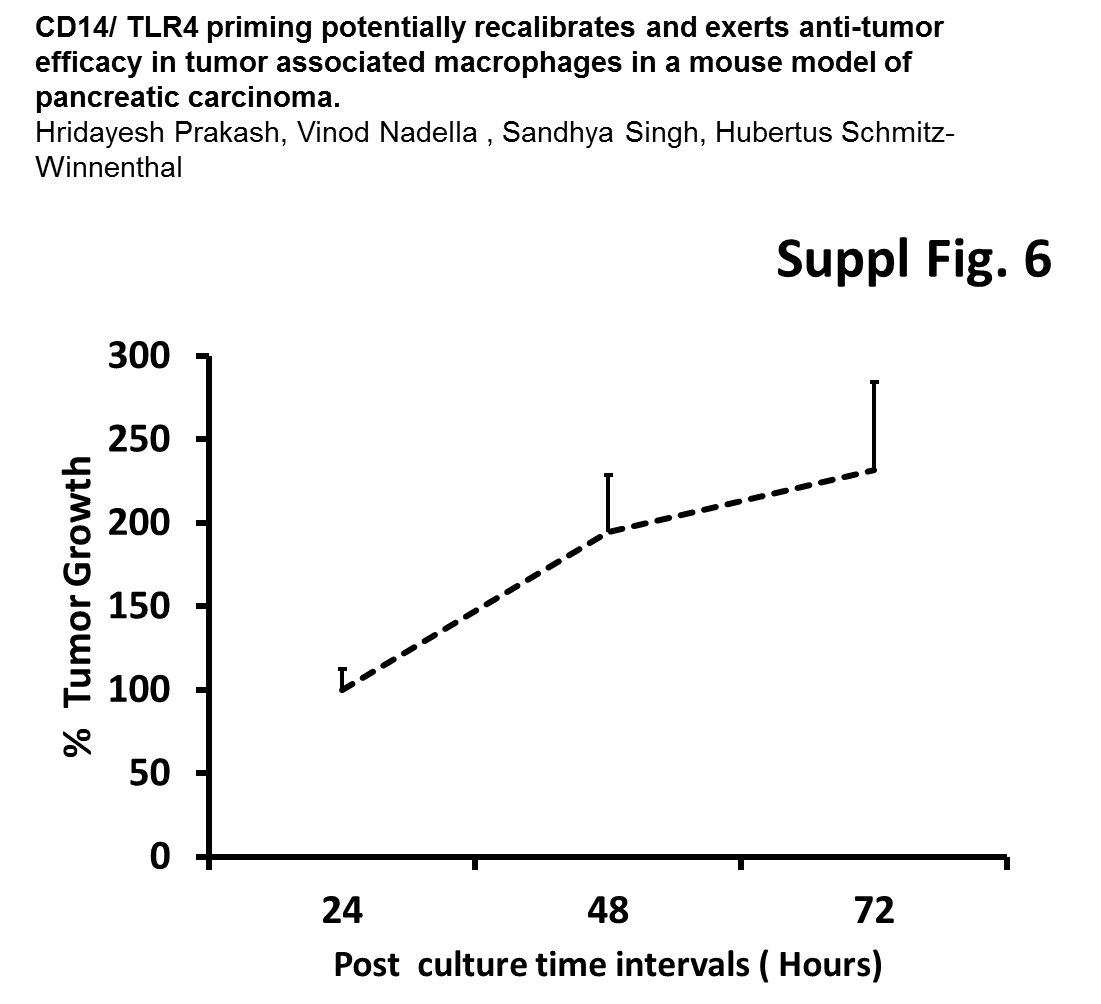


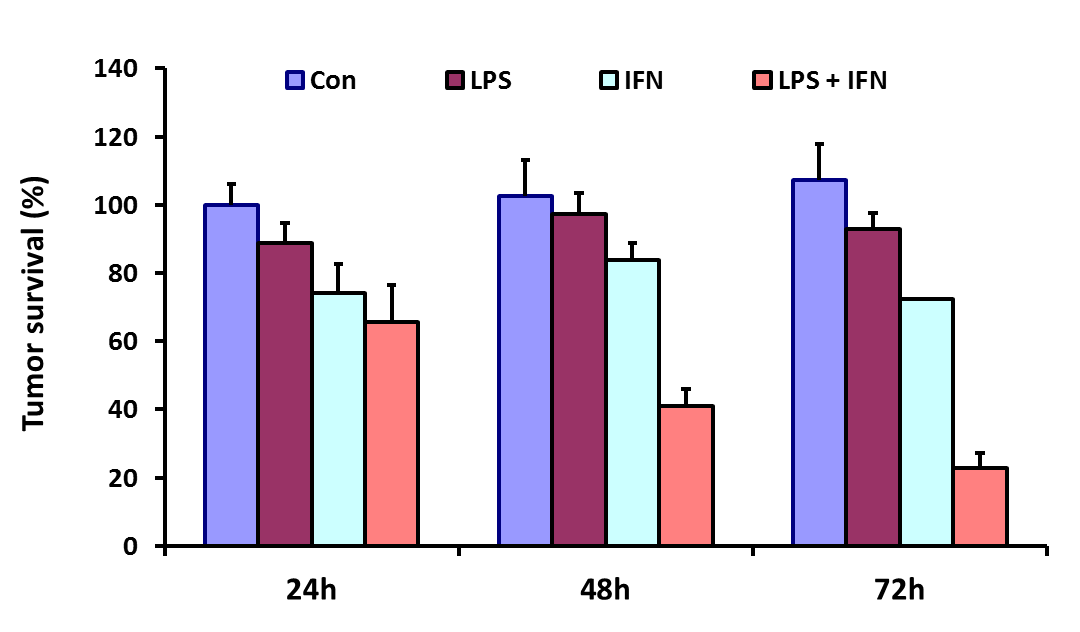


**Suppl Fig. 7**

**CD14/ TLR4 priming potentially recalibrates and exerts anti-tumor efficacy in tumor associated macrophages in a mouse model of pancreatic carcinoma.**

Hridayesh Prakash, Vinod Nadella, Sandhya Singh, Hubertus Schmitz-Winnenthal

*****

*****

******

******


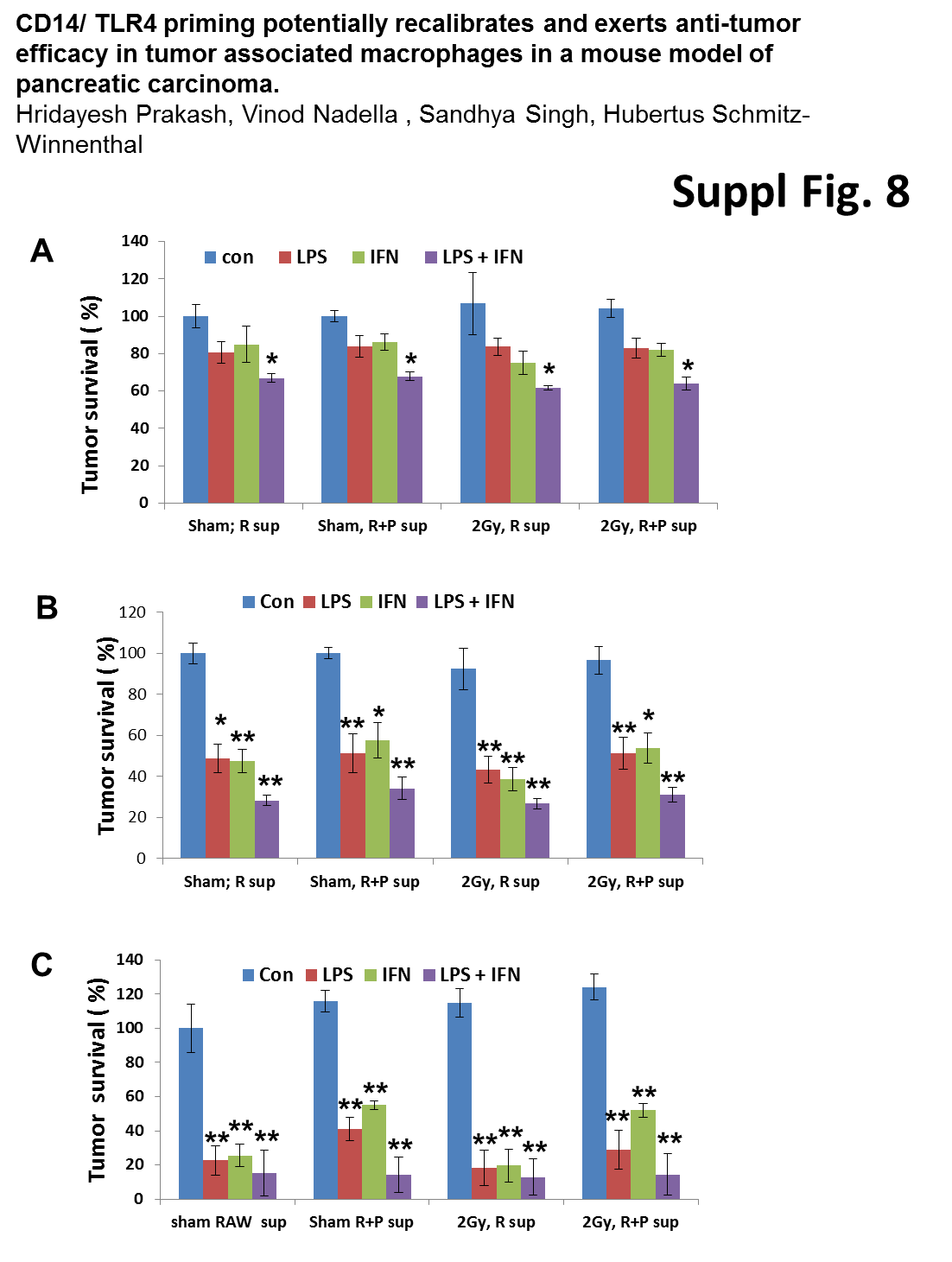


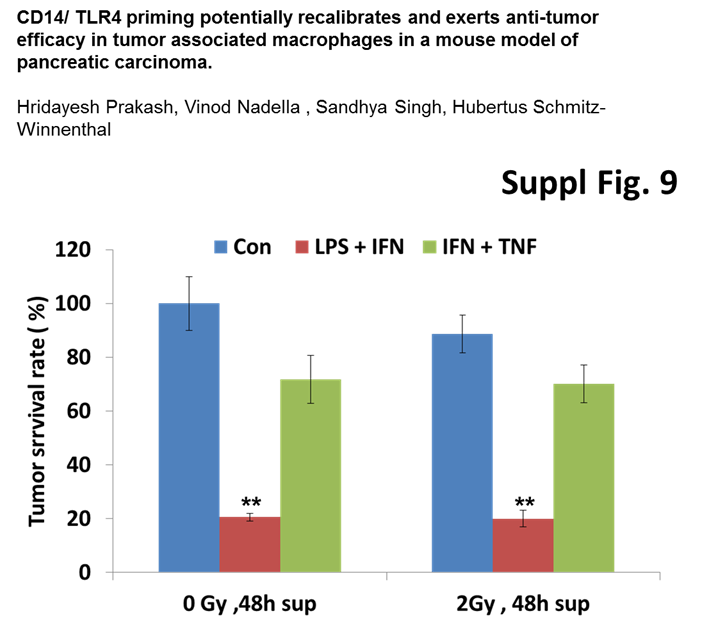

Supplement: Supplementary Information [file srep31490-s1.doc]
